# Supplementary material for: Geology correlates with gut microbial community composition in the Mountainsnails (Oreohelicidae: Oreohelix)
Source: PeerJ. 2025 Oct 7;13:e20080. doi: 10.7717/peerj.20080 (PMC12513373; doi:10.7717/peerj.20080)
Supplement: Supplemental Information 1 [file peerj-13-20080-s001.docx]

**Supplementary Information**

**Table S1**: table showing the outcomes of effect size and power analyses for alpha diversity metrics. Only for Shannon’s Diversity do we have decent effect size and power.

**Table S2**: List of families with significant differential abundances between soil and snail sample types based on ANCOM-BC.

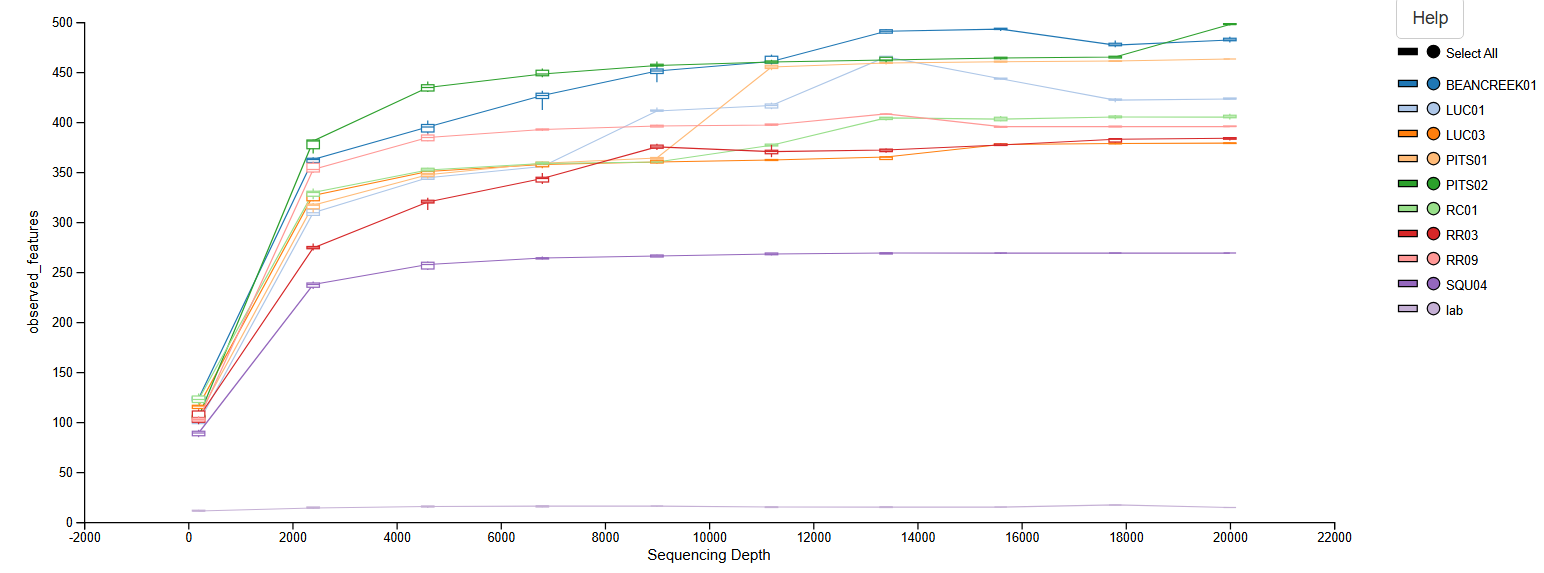
**Figure S1:** Qiime2 rarefaction curves showing a plateau around 4000 reads where more sequencing depth has diminishing returns.


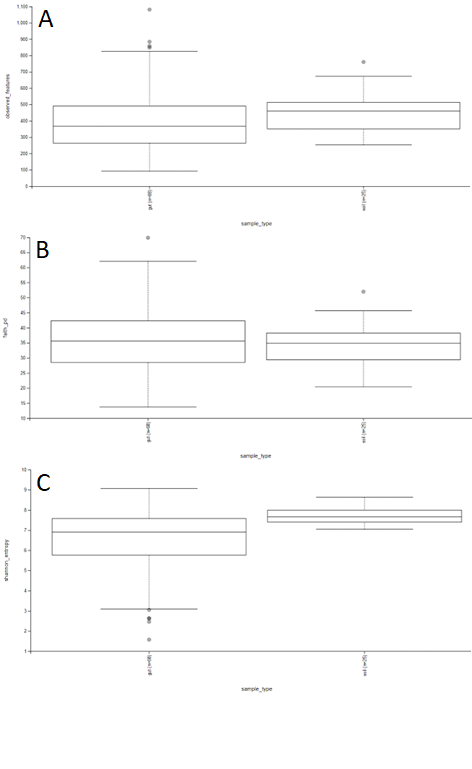
**Figure S2:** Boxplots for alpha diversity differences between sample types (gut on the left, soils on the right). **A.** no difference between sample types for observed features, **B.** no differences between sample types for Faith’s phylogenetic distance, **C.** significant differences between sample types for Shannon’s diversity.
